# Bilateral Endometriotic Cystic Ovaries and Huge Splenic Epithelial Cyst With Elevated CA-125, CA19-9: A Report of a Rare Case

**DOI:** 10.7759/cureus.44256

**Published:** 2023-08-28

**Authors:** Midya R Abdulla, Yadgar A Saeed, Jeza M Abdul Aziz, Syamand A Ahmed, Yadgar A Abdullah, Awat A Sabir, Sivar M Jalal, Aynda S Mohammed, Mohammad D Ali, Mariwan K Rasheed, Muhammad J Rashid, Nguyen Tien Huy

**Affiliations:** 1 Baxshin Research Center, Baxshin Hospital, Sulaymaniyah, IRQ; 2 Biomedical Science Department, Komar University of Science and Technology, Sulaymaniyah, IRQ; 3 Medical Laboratory Department, College of Health, Sulaimani Polytechnic University, Sulaymaniyah, IRQ; 4 Burn Plastic and Reconstructive Surgery Department, Sulaimani Burn Plastic Hospital, Sulaymaniyah, IRQ; 5 College of Medicine, University of Sulaimani, Sulaymaniyah, IRQ; 6 College of Medicine, University of Garmian, Sulaymaniyah, IRQ; 7 Medical Laboratory of Science, College of Health Science, University of Human Development, Sulaymaniyah, IRQ; 8 School of Tropical Medicine and Global Health, Nagasaki University, Nagasaki, JPN

**Keywords:** ca19-9, ca-125, benign tumour, splenic cysts, endometriotic cystic

## Abstract

Histologically benign splenic cysts (SCs) resemble splenic sacs. SCs are rare. Here, we present and discuss a new case of bilateral endometriotic cystic ovaries with massive SCs. A 26-year-old single female visited the hospital with left lower quadrant discomfort and suprapubic pain for three months, accompanied by anorexia, weight loss for these three months, and persistent dysmenorrhea for two years. Splenic examination revealed a soft abdomen with left hypochondria, suprapubic tenderness, and a lump in the upper left quadrant. All laboratory results were normal, except for two cancer antigens (CA-125 and CA 19-9). Therefore, magnetic resonance imaging was used to make the definitive diagnosis, which revealed bilateral ovarian endometrioma with a left upper abdominal cystic mass of splenic origin. When CA-125 and CA-19-9 readings are high, physicians should investigate endometriotic and SCs. Imaging aids diagnosis. Histopathological results are essential. Tools and follow-up should rule out malignancy, and surgery is the best treatment option.

## Introduction

Ovarian endometrioma (OEM) is a benign estrogen-dependent cyst in women of reproductive age. OEMs can cause infertility and subfertility, possibly because of oocyte number and quality. "Chocolate" endometriotic cysts were present in 10% of women aged 25-40 [1]. Endometriosis is expected to affect around 10-15% of females of reproductive age, although this occurrence increases to more than 70% in women with chronic pelvic pain [2]. Rare splenic cysts (SCs) may be parasitic infections, trauma, infarction, congenital, primitive splenic neoplasia, or cystic metastases [3]. SCs are sac-like lesions of the spleen that are histologically mostly benign. SCs are a rare clinical condition, with an incidence of 0.07% in the general population, based on the presence or absence of the cellular epithelial lining. Recently, a link between a true SC and elevated serum levels of cancer antigen 19-9 (CA 19-9) has been observed in a few patients. This association was found in patients who also had high levels of CA 19-9 [4]. The spleen plays an important role in immunomodulation. In cases where increased serum levels of CA19-9 and CA125 are present, it is difficult to differentiate between benign SCs and malignant tumors [5]. However, it is primarily benign and can generally be differentiated from malignant variants using clinical imaging [6]. Here, we report and discuss a new case of bilateral endometriotic cystic ovaries and huge SCs.

## Case presentation

A 26-year-old single female, who lived with her family, visited the hospital with left lower quadrant (LLQ) pain and suprapubic pain for three months, associated with anorexia, weight loss throughout these three months, and chronic dysmenorrhea for two years. There was no history of any chronic disease, any drug use, only analgesics used during the menstrual period, no history of trauma or surgery, no smoking or alcohol consumption, or a family history of malignancy. There was no contact with farm animals.

On clinical examination, she had a soft abdomen, but left hypochondria and suprapubic tenderness, and a mass in the left upper quadrant could be felt on splenic examination.

Her left ovary contained two unilocular thick-wall cystic masses with numerous small papillary projections, and vascularity was only detected in the wall of the largest mass (75×76×60 mm). Her right ovary contained a single unilocular thick-wall cyst measuring 77×60 mm, and her abdominal and pelvic ultrasounds showed a well-defined regular outline solid mass measuring 136×112×114 mm with obvious vascularity and pushed up the spleen. Magnetic resonance imaging (MRI) was requested for further details.

**Figure 1:**
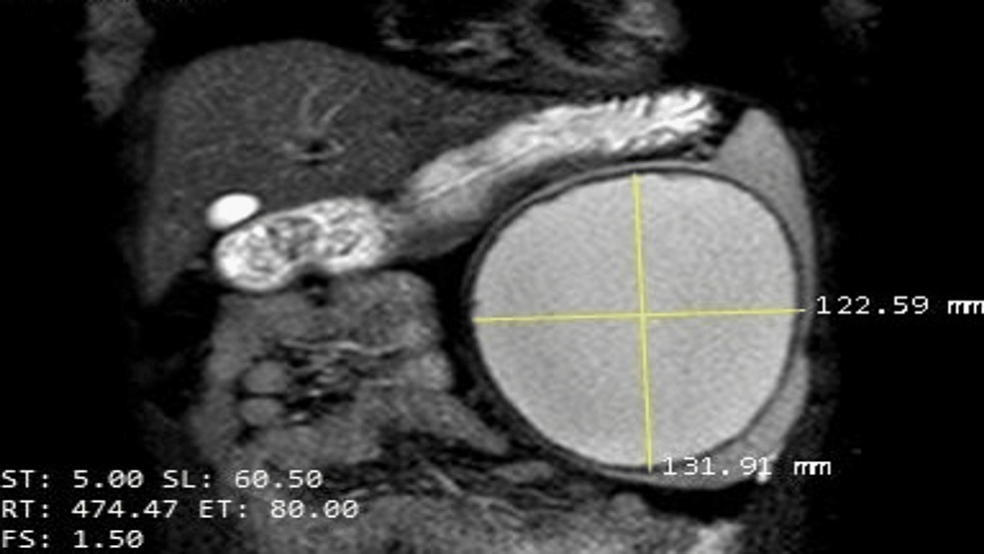
A unilocular cystic mass measuring (13×12 cm) size was seen on T1-, T2-weighted magnetic resonance image (MRI) showing fluid signal intensity within the cyst

MRI revealed a thin-walled unilocular cystic mass measuring 13×12 cm in size in the left upper abdomen on T1- and T2-weighted fluid signal intensity, no blood signals, and no blood diffusion restriction. The post-contrast scan showed marginal enhancement with stretched splenic parenchyma over part of it, which displaced the pancreatic tail superiorly and compressed the left kidney posteriorly; no enhancing solid component was observed (Figure 1).

**Figure 2:**
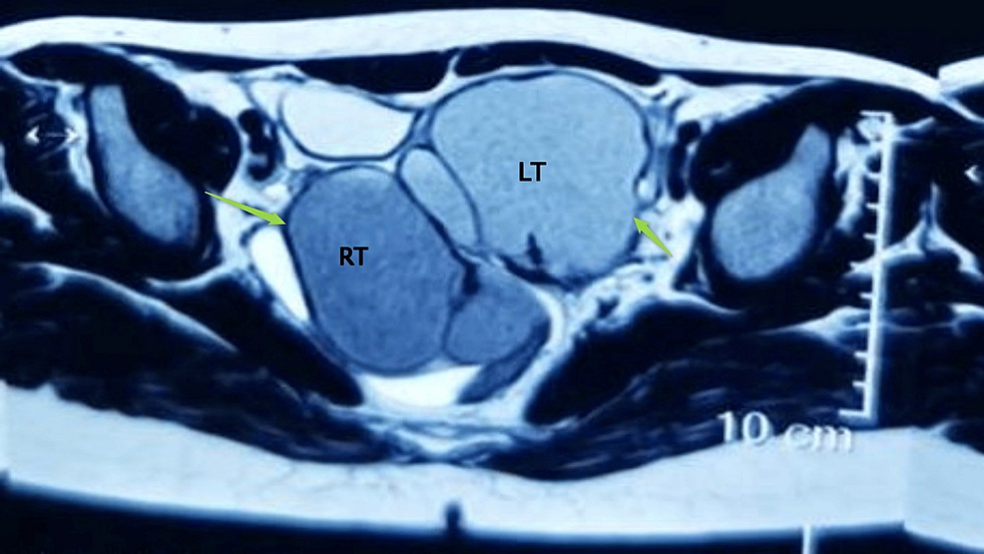
Bilateral ovarian multilobulated cystic mass contains blood measuring right (64×60×65 mm) and left (70×65×60 mm) with high T1 signal, T2 weight low (shading sign)

In addition, a bilateral ovarian multilobulated cystic mass containing blood measuring right (64×60×65 mm) and left (70×65×60 mm), with high T1 signal, T2 weight low (shading sign) with diffusion restriction of the fluid content, both located posterior to the uterus and adjacent to each other (Figure 2) and other findings were unremarkable. MRI suggested bilateral OEM, with a splenic left upper abdominal cystic mass in origin.

Her complete blood count (CBC) tests were unremarkable including RBC=4.55×10^6^/uL, WBC=5.9×10^3^/uL, and hemoglobin (Hb)=13.8 g/dL, and renal function tests were normal in which urea was 20.5 mg/dL and creatinine was 0.72 mg/dL (0.5-0.9 mg/dL). The serum concentrations of both cancer antigens CA-125 and CA19-9 were 204.1U/mL and 70.64 IU/mL, respectively, showing a high concentration of these markers.

A group of general surgeons and gynecologists with 10 years of surgical expertise decided to perform surgical intervention through a midline laparotomy. Each ovary contained a large cyst (6×7 cm). Cystectomy was performed, and the adhesion between the cysts and the posterior uterine wall was removed. After checking bilaterally, ova were present in both ovaries, and good hemostasis was achieved.

**Figure 3:**
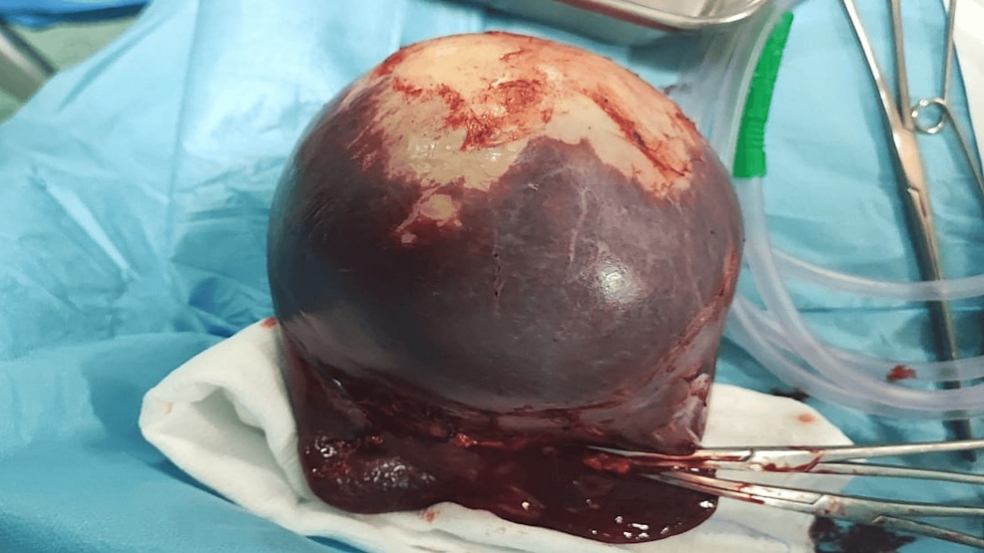
Removed spleen showing a large cystic lesion

In splenic exploration, a very large cyst lesion in the spleen was present, which occupied all spleen parenchyma (Figure 3), so the general surgeon decided to do a splenectomy, hemostasis secured, and corrugate drain left inside the abdomen, then the incision was closed in two layers using a 2-0 Vicryl suture. Excisional biopsy of two large ovarian cysts and splenectomy were performed for histopathological examination. Following surgery, the patient received the following intravenous medications: ceftriaxone 1 g 12 h/day for six days, Flagyl 8 h/day for six days, fluids (2 liters of dextrose normal saline every 24 h for two days), and paracetamol 1 g 12 h/day for six days.

Histological results confirmed that both ovarian sac lesion cysts were hemorrhagic endometriotic cysts with benign behavior. The histology of the splenic lesion shows a huge solitary cyst of the spleen rimmed by a normal attenuated layer of splenic white and red pulp, and most lining of the cyst has been lost through repeated bouts of hemorrhage but there are intact spots where a single layer of cuboidal epithelial cells with benign nuclear features are supported by a thick layer of hyalinized fibrous stroma and organized fibrin.

Following the predetermined postoperative follow-up period, the patient's recovery was smooth and uneventful. Feeding began two days after surgery and continued until regular bowel movements were produced. On the second postoperative day, the patient was discharged without adverse events.

## Discussion

Endometriotic cysts are prevalent benign gynecological disorders linked to high CA-125 and CA-19.9 levels. However, excessively elevated markers and an adnexal mass suggest ovarian cancer, making it difficult to distinguish between the two diagnoses [7]. CA-125 can be helpful as a tumor marker to distinguish between benign and malignant ovarian masses. Furthermore, compared to mucinous tumors, serous tumors showed a stronger correlation with elevated CA-125 levels. The tumor marker CA-125 is now generally acknowledged as a predictive and prognostic factor in CA-125-positive ovarian cancers [7,8]. The most common site of endometriotic lesions is the ovary [9-11]. Ovarian cysts typically develop during pregnancy. Cysts can be benign or malignant [1]. Malignancy must be suspected when the diameter of an endometrial cyst surpasses 15 cm [12]. The current case had a right (64×60×65 mm) and left (70×65×60 mm) ovarian cyst and a histopathologically confirmed benign ovarian cyst of endometriotic origin.

SCs can be primary or secondary. Primary cysts are either parasitic (60%) or non-parasitic. Congenital non-parasitic cysts are secondary cysts. Young people are more likely to develop cysts in the upper pole of the spleen [13-15].

Few cystic malignant splenic tumors have been identified in the literature, and most real SCs are epithelial and are thought to contain embryonic inclusions of epithelial cells from neighboring organs [16]. As in the present study of spleen epithelial cysts, a mass was palpated in the left upper quadrant.

Later, the preferred imaging modalities for diagnosing SCs were US, CT, and MRI [10,17]. These imaging findings and the elevated serum CA19-9 level strongly suggested an epithelial cyst, either epidermoid or lymphoepithelial [18]. Therefore, US was performed on the abdomen, and during screening, a large SC was accidentally found, which was confirmed by MRI.

A previous study proved the correlation between the SC and the tumor marker CA19-9; the elevated level will return to normal after cyst removal [4].

Most patients had no symptoms unless the tumor was large enough to compress the surrounding organ [19]. The patient presented with LLQ pain for three months due to displacement of the pancreatic tail and posterior compression of the left kidney.

Treatment is recommended for symptomatic or complicated large SCs. Large cysts are more likely to rupture and lead to other problems, and previous reports have stated that cysts >5 cm in diameter require surgical treatment [5].

However, partial splenectomy was successful. If the cyst is tiny and one splenic pole has an intact parenchyma, partial splenectomy may be considered. This preserves some splenic function and may minimize overwhelming post-splenectomy infection [20]. Due to cyst rupture and co-existent gallstones in our case, but due to the large size and to avoid a rapturing cyst, laparotomy was selected to remove the spleen in the present study.

## Conclusions

Since elevated levels frequently imply malignancy, the association between tumor markers CA-125 and CA19-9 and these benign conditions complicates differential diagnosis. To make an accurate diagnosis and start the right course of treatment in such cases, a thorough and comprehensive evaluation is essential. Additionally, this case highlights the importance of tumor markers in benign cystic diseases as well as malignancies, giving important new information about their function in clinical practice. When cysts cause symptoms and are >5 cm in size, the recommended treatment is partial or complete splenectomy. US and MRI are effective diagnostic modalities, though surgical intervention is still the mainstay of treatment for such symptomatic and large cysts.
